# The over-35s: early intervention in psychosis services entering uncharted territory

**DOI:** 10.1192/bjb.2018.28

**Published:** 2018-08

**Authors:** Felix Clay, Sophie Allan, Serena Lai, Siona Laverty, Grace Jagger, Cate Treise, Jesus Perez

**Affiliations:** 1 Cambridgeshire and Peterborough NHS Foundation Trust, Cambridge; 2 University of Cambridge, Cambridge; 3 University of East Anglia, Norwich

## Abstract

**Aims and method:**

Following the introduction of new standards for early intervention in psychosis (EIP) in England, EIP services are accepting referrals for people aged 35–65. The Cambridgeshire and Peterborough EIP service (CAMEO) aimed to explore the immediate implications for the service, especially with regards to referral numbers and sources. Data were collected from April 2016 to June 2017.

**Results:**

Referrals for over-35s represented 25.7% of all new referrals. Fifty per cent of referrals for over-35s were accepted onto caseload (40.2% for under-35s). The over-35s were more likely to be referred from acute and secondary mental health services (*P* < 0.01) and had longer durations of untreated psychosis than the under-35s (*P* = 0.02).

**Clinical implications:**

CAMEO has received a significantly higher number of referrals as a result of age inclusivity, with an increased proportion of patients suffering from severe, acute psychotic presentations and existing mental health difficulties. This has implications for service planning.

**Declaration of interest:**

None.

In April 2016, a new Access and Waiting Time Standard for early intervention in psychosis (EIP) services in England^1^ came in to force. At least half of all referrals to EIP services should have access to and commence a National Institute for Health and Care Excellence (NICE)-concordant package of care for psychosis within 2 weeks of referral. Although EIP services had promoted easy access and prompt responses to new referrals since their inception, the new policy offered detail on how to achieve and report the Standard. In addition, the policy, in accordance with NICE recommendations,^2^ provided information about the need to expand current care provision in order to treat those individuals that might be at risk of developing psychosis, and to ensure prompt access and treatment for people with a first-episode psychosis regardless of their age. The effect of age inclusivity on the *modus operandi* of previously youth-oriented services was unknown. In fact, those EIP services that adhered to the new policy in full entered an uncharted territory that, inevitably, would require cultural and structural changes. However, the magnitude of these changes was unclear and not fully informed by previous evidence. For instance, the paucity of studies on the administrative incidence of first-episode psychosis in people aged over 35 assessed in early intervention settings^3^^–^^5^ complicated new workload calculations and commissioning decisions.

The CAMEO EIP service in the Cambridgeshire and Peterborough NHS Foundation Trust (http://www.cameo.nhs.uk) decided to conduct a service evaluation to determine the initial effects of this policy change and assess how well it was achieving its intended aims with this population. In the first instance, CAMEO managers and clinicians were particularly keen to explore the immediate implications for the clinical service, especially with regards to referral numbers and sources for individuals aged over 35 with a suspected first-episode psychosis. This would help generate meaningful information that could drive local decision-making.

## Method

### Setting and data collection

CAMEO is an EIP service that offers management for people aged 14–65 years suffering from first-episode psychosis across Cambridgeshire and Peterborough, UK. CAMEO serves a very diverse population of around 870 000, from the international scientific community in Cambridge city and multicultural population of Peterborough to a large rural base in the Fens.^6^ Referrals of suspected psychosis are accepted from multiple sources, including general practitioners, other mental health services, the third sector, school and college counsellors, relatives and self-referrals.^7^^,^^8^ CAMEO started accepting referrals of people aged over 35 from 1 April 2016.

Data on referral numbers and sources for people aged over 35 were collected over 15 months, from 1 April 2016 to 30 June 2017. Additional personal information was de-identified and did not contain sensitive details. It included data available from the Cambridgeshire and Peterborough NHS Foundation Trust electronic clinical records, such as demographic information (age, gender, ethnicity and marital status) for all referrals, and duration of untreated psychosis (DUP) and working diagnosis (usually based on the clinical judgement of two senior clinicians, including a senior consultant psychiatrist, and discussions at multidisciplinary team meetings) for those referrals accepted onto the CAMEO case-load. For comparative purposes, we also collected some information for under-35s referred to our service during the same period. This included referral numbers and sources, age, gender and DUP for those accepted to case-load.

Data analysis and publication followed the guidelines established by the *Anonymisation Standard for Publishing Health and Social Care Data*.^9^ Raw data were not shared with any third party.

### Statistical analysis

All analyses were performed using version 20 of SPSS (SPSS, Inc., Chicago, Illinois). Comparisons were made using the χ^2^ test for categorical variables and *t*-test or Mann–Whitney U-test for continuous variables. A *P*-value of less than 0.05 represented a significant difference.

## Results

### Referral numbers and characteristics

One hundred and sixty-two referrals for individuals over 35 were received during the 15-month evaluation period; 458 referrals were recorded for people under 35. Thus, referrals for over 35s represented 25.7% of all referrals.

**Fig. 1:**
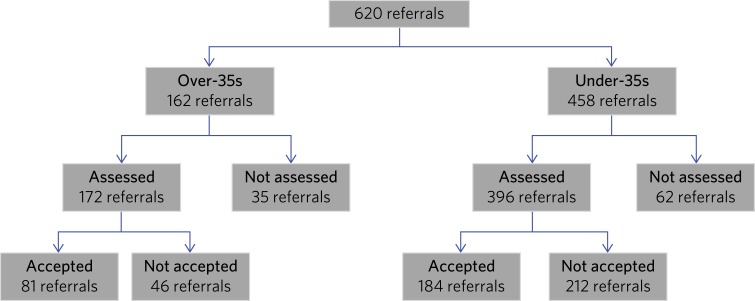
Flow chart for referrals received by CAMEO from April 2016 to June 2017.

50% of referrals for patients over 35 were accepted onto the CAMEO case-load (*n* = 81). Based on the population aged 35–65 in Cambridgeshire and Peterborough,^10^ this represented an administrative incidence of approximately 25 per 100 k per year for this group. Notably, a lower proportion of the total number of referrals for individuals aged under 35 were accepted onto case-load (40.2%) (Fig. 1).

Of the referrals for over-35s, 21.7% (*n* = 35) were not assessed and 28.3% (*n* = 46) were assessed but not accepted onto case-load. Reasons for those over 35 referred but not assessed or taken onto CAMEO were: (a) absence of psychotic symptoms and/or diagnosis of non-psychotic disorder, usually anxiety disorders, after assessment (*n* = 55); (b) evidence of a first-episode psychosis in the past (*n* = 12); and (c) psychotic symptoms in the context of neurodegenerative disorders, i.e. dementia (*n* = 3). The rest were not taken onto case-load for a variety of reasons, such as change of residence to outside CAMEO's catchment area, disengagement during the assessment period or cancellation of referral.

The mean age for all referrals for this group was 47.66 (s.d. = 8.44, range = 36–66.5 years). Fifty-one per cent of referrals for over-35s were female *v.* 38% for under-35s (χ^2^ (4) = 420.55, *P* < 0.01). Forty-four (54.3%) out of the 81 referrals for over-35s finally accepted onto case-load were women.

Approximately one-third of over-35s referred to CAMEO were married or cohabiting, 16.3% were divorced or separated, and 34.7% were single (15.6% not known/recorded). The majority (60.5%) considered themselves White British (the remainder were White other (11.1%), Asian British (1.2%), Asian other (6.8%), African–Caribbean British (1.2%), African–Caribbean other (2.5%), other (3.1%) and not known/recorded (13.6%)).

### Referral sources

**Fig. 2:**
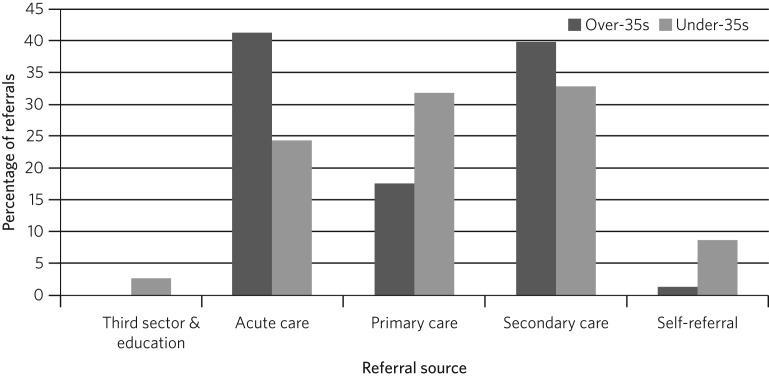
Percentage of referrals for over- and under-35s by referral source.

A higher proportion of referrals for over-35s were received from acute (acute psychiatric wards and crisis resolution home treatment teams) and secondary (community mental health teams) mental health services in comparison with under-35s, who were referred from primary care more often. Differences in referral sources between the two groups were statistically significant (χ^2^ (5) = 27.84, *P* < 0.01). Also, self-referrals from over-35s were less common (2 *v.* 39 individuals) (Fig. 2). Notably, 45.9% of all over-35s referred to our service had a confirmed previous history of mental health problems.

### Duration of untreated psychosis

DUP for over-35s accepted onto case-load ranged from 2 days to 20 years (median 2.6 months, mean = 1.88 years; s.d. = 4.32; *n* = 68, 13 not known). Five patients had a DUP of more than 10 years, and another six of more than 3.5 years. Under-35s had a mean DUP of 8.5 months (s.d. = 1.76 years, median = 1 month, range 1 day to 12 years; *n* = 157, 27 not known). The difference in DUP between under- and over-35s was statistically significant (*u* = 3129.5, *P* = 0.02).

DUP was longer than 3 years for 13.5% of patients over 35 accepted onto case-load (*n* = 11), compared with 3.8% for patients aged under 35 (*n* = 7).

### Working diagnosis

Working diagnoses for the over-35s accepted onto case-load were as follows: unspecified nonorganic psychosis (22.2%, *n* = 18), psychotic depression (16.0%, *n* = 13), delusional disorder (14.8%, *n* = 12), bipolar disorder (12.3%, *n* = 10), schizophrenia (11.1%, *n* = 9) and acute and transient psychotic disorder (8.6%, *n* = 7), with a further 6.2% (*n* = 5) having other diagnoses, including schizoaffective disorder and drug-induced psychosis, and 8.6% (*n* = 7) not known/recorded. Differences in diagnoses by gender were statistically significant (χ^2^ (7) = 14.30, *P* = 0.05); women were more likely to suffer from affective psychoses, such as psychotic depression.

## Discussion

Our findings contribute to a sparse research landscape looking into the administrative incidence of first-episode psychosis in people aged over 35 assessed in EIP settings. What little is so far known has been gathered from services already offering a broader EIP service in predominantly urban areas prior to 2016.^3^^–^^5^ By collecting data after the introduction of the new Access and Waiting Time Standard,^1^ we were able to evaluate the effects of these changes in existing EIP services, such as CAMEO, and anticipate further challenges and opportunities.

Following the changes to our service, almost 26% of new referrals were for patients over 35 years old. This confirms previous evaluations in early-adopter services, which suggested that patients over the age of 35 would make up a significant proportion of referrals, ranging from 25 to 33%.^3^^–^^5^ A higher proportion of patients over the age of 35 were referred from secondary and acute care in comparison with those aged under 35; the over-35s were more likely to have existing mental health issues.

Since referral processes may differ across EIP services, data from early-adopter services are difficult to compare with ours; however, they also suggested relatively few referrals from primary care for this older group. This might reflect lack of awareness in the wider health system, but, for some patients, it may well be related to psychosis developing as a secondary feature of depression and other conditions for which they had already received some support.^11^ Interestingly, 50% of referrals for over-35s were taken on by our early intervention service, whereas only 40.2% of referrals for under-35s were accepted to case-load. This would also support the idea of those aged over 35 suffering from a longer history and higher burden of mental health issues.

Previous studies suggested a different distribution of diagnoses for older *v.* younger patients, with an increased proportion of primarily affective psychoses in over-35s.^3^^,^^4^ Our results reaffirm these findings, with 16% of over-35s suffering from psychotic depression. The proportion of our patients aged over 35 with non-affective psychosis, approximately 55–60%, is similar to that found by previous evaluations and lower than would be expected in younger patients, where non-affective psychosis is usually reported in approximately 75% of cases.^4^ This is consistent with the natural course of mood disorders, such as resistant depression, which becomes progressively more prevalent in older patients, some of whom may have suffered hypomanic episodes for which they did not seek treatment. Non-affective psychotic disorders are less likely to present for the first time over the age of 35.^11^ Also, the higher representation of females in our over-35s sample and those of other studies may reflect a bimodal pattern of psychotic disorders in women, with an first peak at around the same age as in men (18–25 years) and a further peak, usually of an affective nature, in the 40s.^12^ A willingness to treat these older female patients would support age inclusivity across EIP services.

Selvendra *et al*^13^ showed that older patients referred to their mental health service in Italy had been unwell for significantly longer than younger patients. Our results, from an EIP context, also found a statistically significant difference in DUP between over- and under-35s. This indicates the need to continue to enhance outreach approaches to detect emerging psychotic symptoms earlier,^8^ or to consider different approaches for a group of patients whose illness may have become more chronic by the time they are assessed by EIP services.

Although only three referrals were not taken on by CAMEO owing to comorbid dementia in our evaluation, other studies have shown a steady increase in transition to organic pathology in older patients. In fact, neurodegenerative diagnoses creep into the fold as the upper age limit increases above 35.^14^ Accordingly, follow-up studies of over-35s treated in EIP services under the new Standard^1^ will be required in order to evaluate this potential clinical issue and the subsequent effects on services that are not designed to treat such conditions.

In summary, our evaluation has begun to unfold the practical challenges that the implementation of the new Access and Waiting Time Standard^1^ brings to established EIP services with regards to age inclusivity. The CAMEO service received a significantly higher number of referrals as a result of this, with an increased proportion of patients suffering from severe, acute psychotic presentations and with existing mental health issues for which they had already received treatment. These patients were more likely to be referred from secondary mental health services after an acute crisis, and to have longer DUP and psychotic symptoms in the context of other conditions, such as mood disorders.

The main limitation of this evaluation pertains to the collection of data from electronic records routinely employed in clinical practice; some clinical information was missing and working diagnoses were not confirmed with structured diagnostic questionnaires. However, data on referral numbers and sources, which represented the main purpose of this work, were complete and will aid future analyses on clinical and functional outcomes after completion of the early intervention care pathway. This should help to determine whether EIP services, as currently implemented, achieve the required standards with a group of patients whose characteristics clearly differ from those traditionally treated in what used to be exclusively youth-oriented clinical services.

## About the authors

**Felix Clay** is Core Trainee in Psychiatry at CAMEO Early Intervention Services, Cambridgeshire and Peterborough NHS Foundation Trust; **Sophie Allan** is Assistant Psychologist at CAMEO Early Intervention Services, Cambridgeshire and Peterborough NHS Foundation Trust; **Serena Lai** is Consultant Psychiatrist at CAMEO Early Intervention Services, Cambridgeshire and Peterborough NHS Foundation Trust, **Siona Laverty** is Foundation Year 2 Doctor at CAMEO Early Intervention Services, Cambridgeshire and Peterborough NHS Foundation Trust; **Grace Jagger** is Specialty Trainee in Psychiatry at CAMEO Early Intervention Services, Cambridgeshire and Peterborough NHS Foundation Trust; **Cate Treise** is Clinical Psychologist at CAMEO Early Intervention Services, Cambridgeshire and Peterborough NHS Foundation Trust; and **Jesus Perez** is Lead Consultant Psychiatrist at CAMEO Early Intervention Services, Cambridgeshire and Peterborough NHS Foundation Trust, Associate Lecturer at the Department of Psychiatry, University of Cambridge, Honorary Professor of Psychiatry at the University of East Anglia and Lead Director of the National Institute for Health Research (NIHR) Clinical Research Network in the East of England.
